# Red fluorescent *Xenopus laevis*: a new tool for grafting analysis

**DOI:** 10.1186/1471-213X-9-37

**Published:** 2009-06-23

**Authors:** Christoph Waldner, Magdalena Roose, Gerhart U Ryffel

**Affiliations:** 1 Institut für Zellbiologie (Tumorforschung), Universität Duisburg-Essen, Hufelandstr. 55, D-45122 Essen, Germany

## Abstract

**Background:**

Fluorescent proteins such as the green fluorescent protein (GFP) have widely been used in transgenic animals as reporter genes. Their use in transgenic *Xenopus *tadpoles is especially of interest, because large numbers of living animals can easily be screened. To track more than one event in the same animal, fluorescent markers that clearly differ in their emission spectrum are needed.

**Results:**

We established the transgenic *Xenopus laevis *strain tom3 that expresses ubiquitously red fluorescence from the tdTomato gene through all larval stages and in the adult animal. This new tool was applied to track transplanted blastemas obtained after tail amputation. The blastema can regenerate ectopic tails marked by red fluorescence in the host animal. Surprisingly, we also found contribution of the host animal to form the regenerate.

**Conclusion:**

We have established a useful new tool to label grafts in *Xenopus *transplantation experiments.

## Background

The green fluorescent protein (GFP) has successfully been used as a marker gene in the past years. It was also applied to transgenic *Xenopus *either to label grafts (e. g. [1] or cell lineages by using specific promoters (e. g. [2-4]). The GFP derivatives cyan fluorescent protein (CFP) and yellow fluorescent protein (YFP) expanded the color range and were also successfully used in transgenic *Xenopus *[5-7]. Although both markers can be used independently by applying appropriate filter sets, there is some overlap of the emission spectra. Therefore, to track more than one event in the same animal reliably, fluorescent markers that clearly differ in their emission spectrum are needed. The red fluorescent protein DsRed has been isolated from Discosoma sp. to be used as a new tracer with higher wavelength emission, but the long maturation time and poor solubility of the tetrameric DsRed protein [8] prevented its widespread use in transgenic animals. We also observed toxic effects in *Xenopus *of DsRed under the control of the ubiquitously active CMV promoter (MR, unpublished data). Nevertheless, some transgenic animals were made using DsRed in zebrafish [9] or its faster maturating derivatives in Drosophila [10], mice [11] and rats [12]. Progress was achieved a few years ago, when variants of monomeric molecules derived from DsRed were generated giving rise to different color shades of red [13]. All derivatives are characterized by a shorter maturation time and an improved solubility as compared to the wild type DsRed protein, with tdTomato showing the highest brightness and photo stability. To generate a new tool to label grafts we established in the present study a ubiquitous red fluorescent transgenic *Xenopus laevis *strain using the CMV promoter driven tdTomato sequence.

We applied this newly established tool to track cell fate during *Xenopus *tail regeneration. After tail amputation of the tadpole, a mass of proliferative cells is formed underneath the wound epidermis within 24 hours. This mass of cells is commonly called the 'blastema', and is able to regenerate to an imperfect copy of the amputated tail within a few days (reviewed in [14,15]). Although this regenerated tail has less well organized myotomes, the tadpoles are able to swim. Recent studies suggest that V-ATPase dependent proton flux [16] and apoptosis [17] are essential for blastema formation. However, nothing is known whether the blastema generates ectopic tails upon transplantation or whether the interaction with the surrounding amputated tissue is needed. We therefore performed transplantation experiments using the red fluorescent Xenopus strain and followed the fate of the graft as well as of the surrounding host tissue.

## Results

### Establishment of a red fluorescent *Xenopus laevis *strain

**Figure 1:**
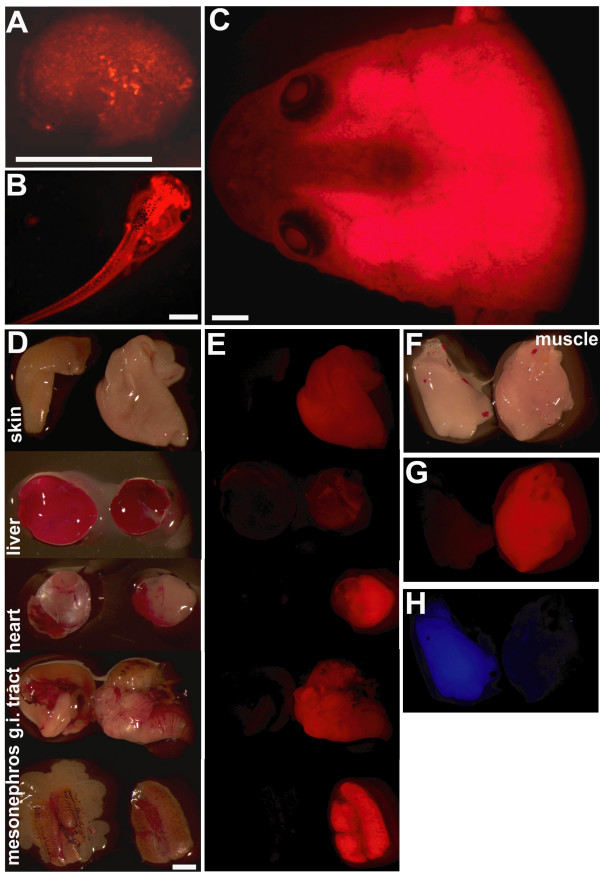
**Transgenic Xenopus laevis strain expressing ubiquitous red fluorescence**. F1 animals of the tom3 strain at the neurula (**A**), larval (**B**) or froglet stage (**C**) seen with the red fluorescence filter set. **D: **Isolated tissue of a control froglet (left) and a froglet of the tom3 strain (right) seen in normal light. **E: **Same tissue samples seen in the red fluorescence filter set. **F-H: **Isolated muscle of a froglet of the blue fluorescent C5 strain [5] (left) and of the red fluorescent tom3 strain (right) seen in normal light (**F**), with red fluorescence filter set (**G**), or blue fluorescence filter set (**H**). Scale bars equal 1 mm.

**Figure 2:**
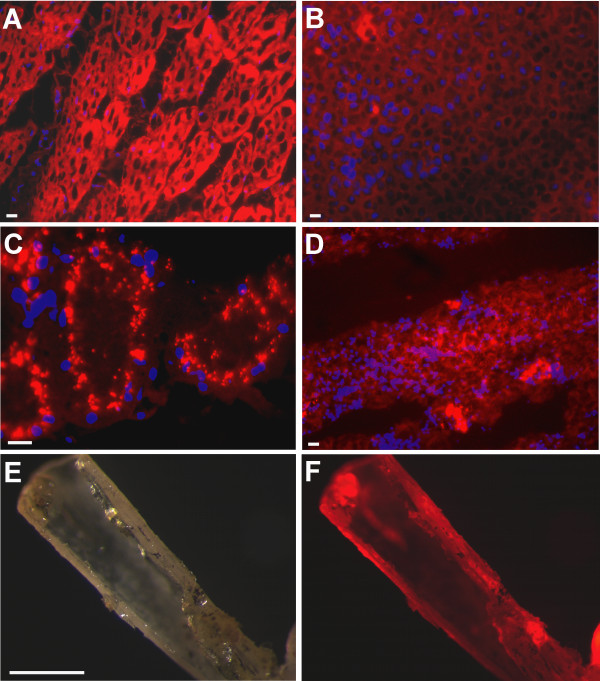
**Adult animals of the tom3 strain express red fluorescence in a variety of tissues**. **A-D: **Cryosections (10 μm) were counterstained with DAPI after methanol fixation (20 min.) to visualize cell nuclei. Overlays were done with AxioVision software using false colours. Note: Due to fixation endogenous red fluorescence as well as the tissue structure suffered. Sections were made from limb muscle (**A**), heart (**B**), kidney (**C**) and liver (**D**). **E-F: **Macrosection of a limb bone seen in normal light (**E**) or with red fluorescence filter set (**F**). Scale bars equal 10 μm (**A-D**) or 1 mm (**E-F**).

To label cells by red fluorescence we have chosen the red fluorescent protein tdTomato controlled by the ubiquitously active CMV promoter. Transgenic animals for the pCSCMV:tdTomato construct were generated. Larvae showing homogenous red fluorescence were selected and grown to sexual maturity. The tom3 founder female could be identified to transmit the active transgene to the next generation resulting in 50% of the offspring expressing red fluorescence. Animals of this strain exhibit strong and homogenous red fluorescent protein expression starting from neurula stage (Figure. 1A). This expression is maintained in the larval (Figure. 1B) and in the froglet stage in all tissues examined (Figure. 1C–G). The red fluorescence can clearly be distinguished from the cyan fluorescent protein (CFP)(Figure. 1F–H). Red fluorescence was also detected on the cellular level in sections from a variety of tissues (Figure. 2).

### Ectopic tail regeneration due to blastema transplantation

**Figure 3:**
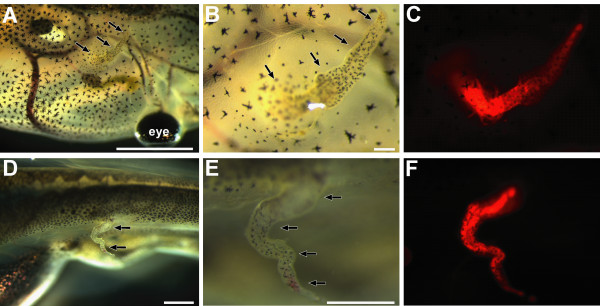
**Ectopic tail formation after blastema transplantation**. 24 hours old blastemas obtained after tail amputation of stage 50 tadpoles of the tom3 strain were transplanted into wild type hosts of the same stage. After 21 days tadpoles were investigated. **A-C: **Tail like outgrowth in the head region: **B: **close-up **C: **red fluorescence filter set. **D-F: **Tail like outgrowth in the trunk: **E: **close-up **F: **red fluorescence filter set. Scale bars equal 1 mm expect in **B **100 μm. The arrows mark the tail-like outgrowth.

**Figure 4:**
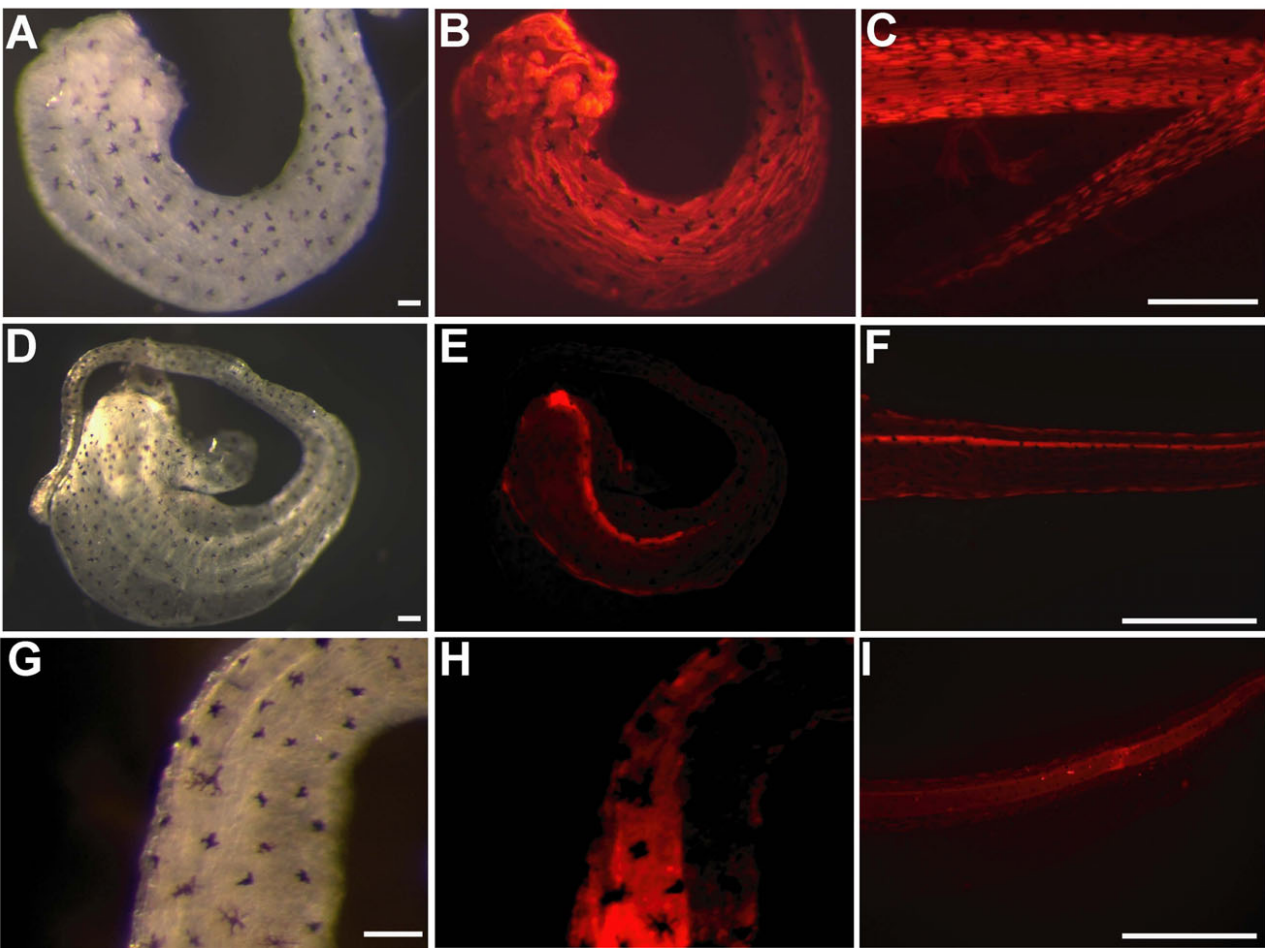
**Tissue identity of blastema derived ectopic tails**. 21 days old ectopic tails or control regenerates (**C**,**F**,**I**) were isolated and immunostained with specific antibodies directed against muscle cells (12/101)(**A-C**), neuronal cells (Xen-1)(**D-F**) or notochord (anti-Coll-2)(**G-I**). Scale bars equal 100 μm in **A**, **D**, **G **or 1 mm in **C**, **F**, **I**. Note, that the red fluorescence reflects the Cy3 labelled secondary antibody exclusively, as the red fluorescence of the tdTomato protein is destroyed by the fixation used for immunostaining.

To investigate the regenerative potential of the tail blastema, transplantation experiments were made using the tom3 strain to track cell fate. The tails of stage 50 larvae of the tom3 strain were amputated. After 24 hours, each newly formed blastema was transplanted under the skin of wild type host animals. We observed outgrowth of a tail-like structure in eight of 25 animals when the blastema was transplanted into the head (Figure. 3A–C) or the trunk (Figure. 3D–F) of the host animal. The regenerate consists of red fluorescent cells (Figure. 3C and 3F) indicating their origin from the transplanted blastema. We did not observe any fluorescent cells elsewhere in the host animal. In the case when no outgrowth was observed, we did not detect any red fluorescent cells at all. To further characterize the regenerated tail-like structure we stained the outgrowth with specific antibodies directed against muscle cells, notochord, or spinal cord (Figure. 4). All three structures are present within the regenerate (Figure. 4B, E, H) and comparable to normal regenerated tails (Figure. 4C, E, I). However, the muscle is poorly segmented, and we did not observe that the tadpoles can move their ectopic regenerate.

**Figure 5:**
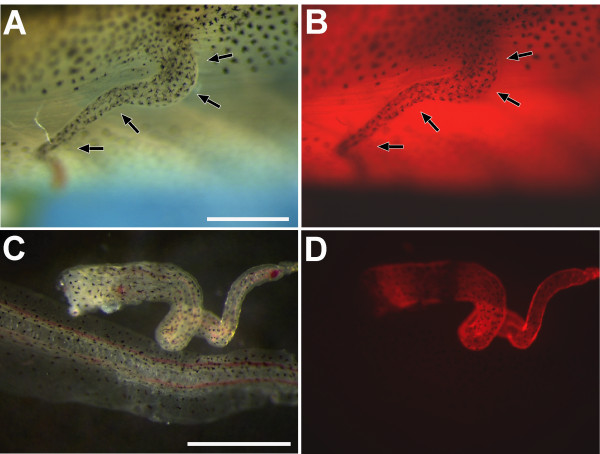
**Contribution of the host animal to blastema derived ectopic tails**. 24 hours old blastemas obtained after tail amputation of stage 50 wild type tadpoles were transplanted into hosts of the tom3 strain. After 21 days tadpoles were investigated. **A-B: **Tail like outgrowth in the trunk shown in the bright field (**A**) or with red fluorescence filter set (**B**). **C-D: **Isolated ectopic tail (upper structure) together with wild type tail shown in normal light (**C**) or with red fluorescence filter set (**D**). Scale bars equal 1 mm. The arrows mark the tail-like outgrowth.

To investigate whether cells of the host animal also contribute to ectopic tail regeneration, we used the tom3 strain to track these cells in transplantation experiments. We transplanted 24 hours old blastemas of wild type larvae into host animals of the tom3 strain. This led to outgrowth of a regenerate in 15 of 46 transplanted animals (Figure. 5A), but fluorescence of this regenerate could not be easily judged in the background of the strong red fluorescence of the host animal (Figure. 5B). Therefore, we isolated the regenerate to monitor fluorescence. Surprisingly, we observed in all ectopic regenerates cells of the host animal labeled by red fluorescence possibly located in the epidermis (Figure. 5D). We thus conclude that the ectopic regenerate is partly derived from the host animal.

## Discussion

In this study we established the red fluorescent transgenic *Xenopus *strain tom3 expressing the CMV promoter driven tdTomato sequence. This is in contrast to the use of DsRed as a transgene that is not compatible with normal development and thus precludes the establishment of a transgenic strain (MR, unpublished data). We could recently confirm the value of tdTomato as stable transgene to mark a *Xenopus *strain containing a functional Cre recombinase [18]. Adult animals of the tom3 strain show ubiquitous and homogenous expression of the red fluorescent protein indicating that the CMV promoter can be used to drive transgene expression also at later stages of development. Therefore, the CMV promoter may be equivalent to the murine ROSA26 promoter that has been proposed for widespread and persistent transgene expression in *Xenopus laevis *[19]. This transgenic strain can thus be used to label grafts isolated at any time point in development. Animals of the tom3 strain showing ubiquitous and strong expression of tdTomato developed absolutely normal suggesting that tdTomato has a high biocompatibility as proposed for a recently engineered noncytotoxic DsRed variant, which has the drawback of a reduced brightness [20]. When we transplanted labelled blastemas obtained after tail amputation we found that cells marked by red fluorescence can form an ectopic regenerate, but do not migrate into the host tadpole. We even observed ectopic regeneration in the head pointing to a strong degree of self organization of the blastema. As an orientation of the blastema used for transplantation was not possible, we cannot exclude that transplants not properly orientated failed to outgrow. The regenerate contains the typical tail structures including muscle, spinal cord and notochord. This implies that the blastema formed 24 hours after amputation contains precursor cells to all these tissues and that their differentiation can proceed autonomously of the amputated stump. However, we cannot exclude that the transplanted blastema contained part of the terminal bulb of the spinal cord as well as of the bullet-shaped notochord cells that partially extend into the regeneration bud [21]. The fact that melanophores were present in all regenerates is consistent with a previous study [22] showing that these cells are derived from neural crest precursors and further strengthens the concept of cell recruitment by the blastema. In all cases the ectopic tail lacks the broad tail fin present in the in situ regenerate (data not shown). Possibly the supportive structure of the stump is needed for the formation of the tail fin. Furthermore, the ectopic tail is less well organized lacking proper segmentation and typically with a twisted appearance compared to the normal regenerate. This is reminiscent to the twisted tail regenerates observed upon spinal cord ablation in *Xenopus *[23]. We assume that this deficiency in proper morphology but also the inability for movement of the ectopic tail are possibly due to the lack of a connection to the spinal cord of the host animal.

After tail amputation in *Xenopus *a wound epidermis is formed by migrating cells within the first 24 hours. It is proposed that this wound epidermis signals to the underlying cells to form the blastema (reviewed in [14,15]). Our experiments using labelled host cells expand this model, because our data reveal that the transplanted blastema also recruits cells of the host to form the regenerate. The tissue identity of these cells as well as the underlying mechanism have to be determined in future studies.

## Conclusion

In summary, the newly established red fluorescent tom3 strain offers a powerful tool to further investigate the process of regeneration in Xenopus. In combination with the existing green and yellow [1,7] fluorescent strains, three different cell types can now be labelled in transplantation experiments.

## Methods

### Plasmids

The tdTomato sequence was isolated from pRSET-BtdTomato [13] as a *BamHI/EcoRI *fragment and cloned into pBluescriptIISK+. From the obtained plasmid the *BamHI/XhoI *fragment was isolated and subcloned into pCSGFP2 [3] replacing the GFP2 sequence to generate pCSCMV:tdTomato.

### Transgenesis

The original protocol using restriction enzyme mediated integration [24] was modified by using frozen sperm nuclei and omitting the egg extract to get more normal developing larvae [5]. Individual founders were marked as described [25]. The pCSCMV:tdTomato construct was digested by *SalI *and the fragment lacking the bacterial DNA was purified for transgenesis. Fluorescence microscopy was done with a Leica MZ/FLIII or a Zeiss Axioplan stereomicroscope with the appropriate filters as described [26]. *Xenopus *stages are as defined [27].

### Blastema transplantation

About 1 cm of the tail of stage 50 tadpoles was amputated. The tadpoles were kept in fresh water for 24 hours. The newly formed blastema was cut off and introduced under the skin of the host tadpole by using sharp forceps.

### Immunohistochemistry

The tissue was fixed and stained with the monoclonal antibodies Xen1 (3B1), 12/101, or anti-Collagen type II (Clone II-4CII, MP Biomedicals) essentially as described [28].

## Authors' contributions

CW and GUR designed research. MR made the transgenic Xenopus strain. CW performed transplantation experiments and wrote the paper. All authors read and approved the final manuscript.
